# Vascular resection for locally advanced pancreatic ductal adenocarcinoma: analysis of long-term outcomes from a single-centre series

**DOI:** 10.1186/s12957-021-02238-x

**Published:** 2021-04-18

**Authors:** Claudio F. Feo, Giulia Deiana, Chiara Ninniri, Giuseppe Cherchi, Paola Crivelli, Alessandro Fancellu, Giorgio C. Ginesu, Alberto Porcu

**Affiliations:** 1 Unit of General Surgery 2, Department of Medical, Surgical and Experimental Sciences, University of Sassari, Viale San Pietro 43, 07100 Sassari, Italy; 2 Unit of Radiology, Department of Medical, Surgical and Experimental Sciences, University of Sassary, Viale San Pietro 10, Sassari, 07100 Italy

**Keywords:** Pancreatic ductal adenocarcinoma, Pancreatic surgery, Venous infiltration, Arterial infiltration, Prognosis, Survival

## Abstract

**Background:**

Pancreatic ductal adenocarcinoma (PDAC) is an aggressive malignancy with poor prognosis. Radical surgery is the best option for cure and, nowadays, it is performed by many surgeons also in cases of vascular infiltration. Whether this aggressive approach to a locally advanced PDAC produces a survival benefit is under debate. Most data in the literature come from retrospective comparative studies; therefore, it is still unclear if such an extensive surgery for an advanced cancer is justified.

**Methods:**

A retrospective review of patients with PDAC treated at our institution over a 12-year period was performed. Data concerning patients’ characteristics, operative details, postoperative course, and long-term survival were retrieved from prospective databases and analysed. Factors associated with poor survival were assessed via Cox regression analysis.

**Results:**

A total of 173 patients with PDAC were included in the analysis, 41 subjects underwent pancreatectomy with vascular resection for locally advanced disease, and in 132 patients, only a pancreatic resection was undertaken. Demographics, major comorbidities, and tumour characteristics were similar between the two groups. Length of surgery (*P*=0.0006), intraoperative blood transfusions (*P*<0.0001), and overall complications (*P*<0.0001) were significantly higher in the vascular resection group. Length of hospital stay (*P*=0.684) and 90-day mortality (*P*=0.575) were comparable between groups. Overall median survival (*P*= 0.717) and survival rates at 1, 3, and 5 years (*P*=0.964, *P*=0.500, and *P*=0.445, respectively) did not differ significantly between groups. Age ≥70 years and postoperative complications were independent predictors of lower survival.

**Conclusions:**

Our study confirms that pancreatectomy with vascular resection for a locally advanced PDAC is a complex operation associated with a significant longer operating time that may increase morbidity; however, in selected patients, R0 margins can be obtained with an acceptable long-term survival rate. Older patients are less likely to benefit from surgery.

## Introduction

Pancreatic ductal adenocarcinoma (PDAC) is a very aggressive malignancy with poor long-term prognosis. About 80–85% of patients have a locally advanced disease at diagnosis [1, 2] and the 5-year survival rate is approximately 5–7% [3, 4]. Radical surgical resection is the best option for cure; however, the presence of major vessel invasion is usually considered a criterion for unresectability.

According to the National Comprehensive Cancer Network (NCCN) guidelines [5], a tumour involving the arterial and/or the portal/mesenteric axis is considered a borderline resectable pancreatic cancer (BRPC). However, this is an intermediate category including locally advanced tumours that ranges from resectable to unresectable lesions [6–8]. The vessels involved and tumour extension into the vessel wall may vary consistently; therefore, vascular resection and reconstruction may be achieved by different methods: partial resection with direct closure of the defect, segmental resection with end-to-end anastomosis, segmental resection with interposed venous/artificial graft, or resection and reconstruction of multiple vessels [9, 10]. A recent international consensus statement [11] on the definition of BRPC has considered, besides the anatomic relationship between tumour and vessels, also biological and conditional dimensions. Biological features included suspicion for distant or lymph node metastases, whereas conditional factors comprised patient’s performance status.

Improvements in the perioperative care have resulted in comparable mortality and morbidity rates between resectable and BRPC patients [12]. Venous (portal vein, superior mesenteric vein) infiltration is encountered more frequently than arterial (hepatic artery, celiac trunk, superior mesenteric artery) invasion; nonetheless, R0 resection may be achieved by an experienced hepatobiliary surgeon in both cases. Whether this aggressive surgical approach to a locally advanced PDAC produces a long-term survival benefit is still unclear.

The aim of our study was to compare the oncologic outcomes of patients with locally advanced PDAC who did and those who did not undergo vascular resection. The prognostic factors were also analysed to determine which features were associated with a poor prognosis.

## Methods

### Study population

Data on all pancreatic resections for diagnosis of PDAC performed in our academic hospital from January 2008 to January 2020 were collected from a prospectively maintained database. Vascular resection (venous and/or arterial) was performed when tumour infiltration was suspected based on the radiologic and/or intraoperative evaluation. Demographic, preoperative, intraoperative, and postoperative data were retrieved and analysed. Patients’ characteristics included age, sex, body mass index (BMI), American Society of Anesthesiologists (ASA) score [13], comorbidities, and tumour stage. Preoperative abdominal computed tomography (CT) scan of each patient who underwent vascular resection was reviewed by a radiologist to determine the degree of tumour contact between the tumour and the vessel, less or more than 180°.

The study was approved by our institutional review board, and informed consent was obtained from all patients prior to each procedure.

### Surgical technique

All procedures were performed by the same skilled hepatobiliary surgeon. Depending on the tumour location, a standard Whipple or Traverso-Longmire pancreaticoduodenectomy, a distal splenopancreatectomy, or a total pancreatectomy was performed in all patients. A Roux-en-Y jejunal loop reconstruction was routinely undertaken with an end-to-end telescopic pancreaticojejunostomy and an end-to-side hepaticojejunostomy, both protected by internal drainages. Vascular resection was performed en-bloc with the pancreas when needed to obtain an R0 margin. Vessel reconstruction was undertaken with either a direct running suture, an autologous venous patch (great saphenous vein), or interposition of an autologous venous segment (superficial femoral vein) depending on the length of the resected vessel. All patients received intravenous heparin prior to vessel clamping and then prophylactic subcutaneous low-molecular-weight heparin for 1 month from the operation. Intraabdominal drainages were routinely placed near either the pancreatico-enteric or the bilio-enteric anastomoses.

### Histopathological examination

Intraoperative frozen sections were performed on the hepatic duct and the pancreatic stump in all cases, and resection was extended until a negative margin could be achieved or the operation was changed to a total pancreatectomy. Definitive histopathological reports were reviewed to determine the TNM (Tumour-Node-Metastasis) stage, according to the American Joint Committee on Cancer 7th edition [14]. Pathological confirmation of vessel wall infiltration was routinely performed in all cases of vascular resection. Data on tumour size, lymph node involvement, and perineural and lymphovascular invasion were retrieved and analysed.

### Follow-up protocol

Postoperative complications were graded according to the Clavien-Dindo classification [15]. Definitions from the International Study Group of Pancreatic Surgery (ISGPF) [16–18] were used to evaluate specific complication of pancreas surgery, like postoperative pancreatic fistula (POPF), delayed gastric emptying, and haemorrhage. Mortality was calculated at 90 days from the surgical operation. Follow-up data were collected by an oncologist. All patients underwent clinical, laboratory, and imaging tests at 3, 6, and 12 months postoperatively. Oncological outcomes were obtained from electronic hospital records.

### Statistical analysis

Continuous variables were expressed as mean and standard deviation or median and interquartile range (IQR). Categorical variables were reported as count and percentages. Chi-square test, Student’s *t*-test, or Mann-Whitney *U*-test was used as appropriate, and a *P* value up to 0.05 was considered statistically significant. Survival curves were determined using the Kaplan-Meier method including the log-rank test. The Cox regression model was applied for uni- and multivariate analyses to identify features associated with worst outcomes. Statistical analyses were performed by using SPSS 20 for Windows software (IBM Corp, Armonk, NY, USA).

## Results

From January 2008 to January 2020, a total of 173 patients underwent pancreatic resection for PDAC at our institution. There were 89 females, mean age 68.7 years (range 23–87). In 41 (23.7%) cases, a vascular resection (VR+) was necessary whereas in the remaining 132 subjects (76.3%) no vessel resection (VR−) was performed. Of the 41 patients in the VR+ group, 37 underwent isolated venous resection, 2 isolated arterial resection, and 2 combined venous/arterial resection.

Patients’ details are presented in Table 1. There were no significant differences between groups in demographics, major comorbidities, and tumour characteristics. In the VR+ group, obstructive jaundice was more frequent (*P*=0.001) and a greater number of patients underwent preoperative biliary drainage (*P*=0.003). Abdominal CT scan review of the 41 VR+ patients showed the presence of tumour abutment greater than 180° in 24.4% of cases.

**Table 1:** Patients’ characteristics

	Overall (*n*=173)	VR− group (*n*=132)	VR+ group (*n*=41)	*P*-value
Age (years), mean	68.7±10.8	68.6±11.1	69±10.0	0.850
Male/female	84/89	67/65	17/24	0.298
BMI (kg/m^2^), mean	25.9±3.9	26.6±3.9	23±3.8	0.063
ASA class				
I–II (%)	85 (49.1)	61 (46.2)	24 (58.5)	0.168
III (%)	88 (50.9)	71 (53.8)	17 (41.5)	0.168
Comorbidities				
Diabetes (%)	24 (13.9)	16 (12.1)	8 (19.5)	0.232
Cardiovascular disease (%)	25 (14.5)	20 (15.2)	5 (12.2)	0.638
COPD (%)	2 (1.2)	2 (1.5)	0	0.428
Obstructive jaundice (%)	43 (24.9)	25 (18.9)	18 (43.9)	**0.001**
Preoperative biliary drainage (%)	20 (11.6)	10 (7.6)	10 (24.4)	**0.003**
Tumour stage				
I (%)	47 (27.2)	39 (29.5)	8 (19.5)	0.207
II (%)	70 (40.5)	55 (41.7)	15 (36.6)	0.563
III (%)	47 (27.2)	32 (24.2)	15 (36.6)	0.121
IV (%)	9 (5.2)	6 (4.5)	3 (7.3)	0.485
Degree of tumour contact at CT				
< 180° (%)	31 (17.9)	–	31 (75.6)	
> 180° (%)	10 (5.8)	–	10 (24.4)	

*BMI* body mass index, *ASA* American Society of Anesthesiologists, *COPD* chronic obstructive pulmonary disease, *CT* computed tomography

Operative and postoperative data are summarised in Table 2. Length of surgery was significantly longer in the VR+ group (343.9 versus 295.1 min, *P* = 0.006), but no significant difference between groups regarding the type of operation performed except for total pancreatectomies (*P*=0.005). Duration of ICU stay (1.4 versus 0.7 days, *P*=0.187) and length of hospital stay (18.3 versus 18.9 days, *P*=0.684) were comparable in the two groups. Intraoperative blood transfusions (53.7 versus 19.8%) and overall complications (61 versus 23.5%) were significantly higher in the VR+ cases (*P*<0.0001, in both cases). A POPF occurred in 9.8% and 11.4% of the VR+ and VR− patients, respectively (*P*=0.774). Delayed gastric emptying (7.3 versus 1.5%), postoperative haemorrhage (14.6 versus 2.3%), and reoperation (14.6 versus 5.3%) were observed more frequently in the VR+ patients as compared to the VR− cases (*P*=0.053, *P*=0.002, and *P*=0.048, respectively). Mortality at 90 days from the operation was 14.6 and 11.4% in the VR+ and VR− groups, respectively (*P*=0.575).

**Table 2:** Operative and postoperative details

	Overall (*n*=173)	VR− group (*n*=132)	VR+ group (*n*=41)	*P*-value
Operative time (minutes), mean	309.2±93.2	295.1±96.3	343.9±75.6	**0.006**
Type of operation				
Whipple PD (%)	53 (30.6)	43 (32.6)	10 (24.4)	0.321
Traverso-Longmire PD (%)	65 (37.6)	52 (39.4)	13 (31.7)	0.375
Distal splenopancreatectomy (%)	25 (14.5)	20 (15.2)	5 (12.2)	0.638
Total pancreatectomy (%)	30 (17.3)	17 (12.9)	13 (31.7)	**0.005**
Type of vascular resection				
Venous (%)	37 (21.4)	–	37 (90.2)	
Arterial (%)	2 (1.2)	–	2 (4.9)	
Arteria/venous (%)	2 (1.2)	–	2 (4.9)	
Type of vessel reconstruction				
Direct suture (%)	32 (18.5)	–	32 (78)	
Autologous venous patch (%)	1 (0.6)	–	1 (2.4)	
Autologous venous segment (%)	8 (4.6)	–	8 (19.5)	
Intraoperative blood transfusion (%)	48 (27.7)	26 (19.7)	22 (53.7)	**< 0.0001**
ICU stay (days), mean	1±2.3	0.7±1.6	1.4±3.0	0.187
Clavien-Dindo class				
I (%)	10 (26)	7 (28)	3 (19.5)	0.629
II (%)	30 (17.3)	15 (11.4)	15 (36.6)	0.000
III (%)	14 (8.1)	8 (6.1)	6 (14.6)	0.079
IV (%)	2 (1.2)	1 (0.8)	1 (2.4)	0.379
Overall complications	56 (32.4)	31 (23.5)	25 (61)	**< 0.0001**
Pancreas-specific complications				
POPF (%)	19 (11)	15 (11.4)	4 (9.8)	0.774
Haemorrhage (%)	9 (5.2)	3 (2.3)	6 (14.6)	**0.002**
Delayed gastric emptying (%)	5 (2.9)	2 (1.5)	3 (7.3)	0.053
Reoperation (%)	13 (7.5)	7 (5.3)	6 (14.6)	**0.048**
Hospital stay (days), mean	18.6±12.6	18.9±14.0	18.3±10.5	0.684
90-day mortality (%)	21 (12.1)	15 (11.4)	6 (14.6)	0.575

*PD* pancreaticoduodenectomy, *ICU* intensive care unit, *POPF* postoperative pancreatic fistula

**Fig. 1:**
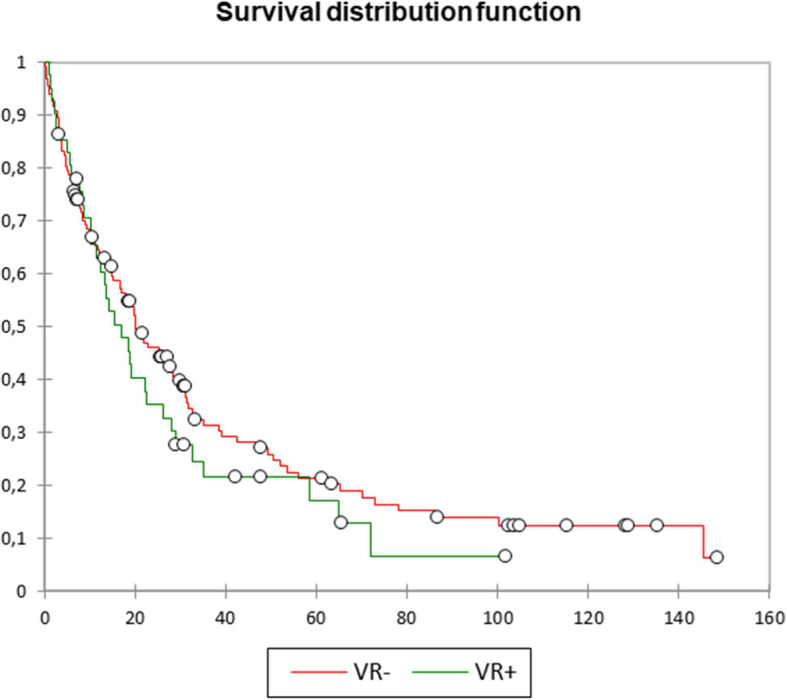
Kaplan-Meier survival curves. Overall survival (*P*=0.717), VR− (red line) vs. VR+ (green line) group

Oncological outcomes are shown in Table 3 and Fig. 1. Mean tumour size (3.5 versus 2.8 cm) and perineural infiltration (61 versus 43.2%) were significantly greater in the VR+ cases (*P*=0.005 and *P*=0.046, respectively). However, lymphovascular invasion (29.3 versus 18.2%, *P*=0.127), lymph node ratio (0.14 versus 0.13, *P*=0.795), and positive resection margins (9.8 versus 6.8%, *P*=0.533) did not differ significantly between groups. Pathological confirmation of vessel wall infiltration was found in 32 of the 41 (78%) VR+ cases. Overall median survival was 15.4 versus 18.4 months in the VR+ and VR− patients, respectively (*P*= 0.717). Median survival in the 37 patients who underwent isolated venous resection was 17 months, whereas in the 4 patients who underwent arterial resection (2 arterial + 2 arterial/venous) it was 5 months (*P*=0.180). Survival rates at 1, 3, and 5 years were 61, 17.1, and 9.8% in the VR+ group, and 61.4, 22, and 14.4% in the VR− patients, respectively (*P*=0.964, *P*=0.500, and *P*=0.445, respectively).

**Table 3:** Oncological data

	Overall (*n*=173)	VR− group (*n*=132)	VR+ group (*n*=41)	*P*-value
Tumour size (cm), mean	3.1±1.2	2.8±1.0	3.5±1.2	**0.005**
Lymphovascular invasion (%)	36 (20.8)	24 (18.2)	12 (29.3)	0.127
Perineural invasion (%)	82 (47.4)	57 (43.2)	25 (61)	**0.046**
Lymph node ratio, mean	0.14±0.18	0.14±0.19	0.15±0.14	0.795
Positive resection margins (%)	13 (7.5)	9 (6.8)	4 (9.8)	0.533
Pathological vessel infiltration (%)	32 (18.5)	–	32 (78)	
Survival				
Overall (months), median	17.7 (0.1–148.6)	18.4 (0.1–148.6)	15.4 (0.9–101.7)	0.717
1 year (%)	106 (61.3)	81 (61.4)	25 (61)	0.964
3 years (%)	36 (20.8)	29 (22)	7 (17.1)	0.500
5 years (%)	23 (13.3)	19 (14.4)	4 (9.8)	0.445

At multivariate analysis (Table 4), age ≥70 years (HR 1.59, 95% CI 1.05–2.43) and postoperative complications (HR 2.08, 95% CI 1.20–3.60) were independent predictors of lower survival. Tumour size ≥ 3cm, lymphovascular invasion, perineural infiltration, positive resection margins, and vascular resection were not predictors of overall survival.

**Table 4:** Uni- and multivariate analysis

	Univariate Patients (MST) *P*-value	Multivariate HR (95% CI)
Age ≥ 70 years	90 (30.03) **0.011**	**1.59 (1.05–2.43)**
Male gender	84 (33.96) 0.939	1.39 (0.90–2.13)
Whipple PD	53 (28.49) 0.399	1.32 (0.86–2.05)
Postoperative complications	56 (20.30) **0.009**	**2.08 (1.20–3.60)**
Tumour size ≥ 3 cm	36 (38.49) 0.223	1.34 (0.87–2.07)
Lymphovascular invasion	36 (37.09) 0.481	0.81 (0.44–1.47)
Perineural invasion	82 (30.13) 0.545	1.47 (0.89–2.43)
Positive resection margins	13 (26.62) 0.512	0.59 (0.26–1.32)
Vascular resection	41 (25.52) 0.311	0.83 (0.48–1.45)

*PD* pancreaticoduodenectomy, *MST* median survival time (months), *HR* hazard ratio, *CI* confidence interval

## Discussion

Previous studies have shown that pancreaticoduodenectomy (PD) with vascular resection is associated with a greater tumour size, higher R1 resection rate, more frequent perineural or lymph node infiltration, and worse survival [12, 19]. However, in other comparative reports, these poorer outcomes were not observed [20–24], and a definitive conclusion cannot be made. In recent years, many surgeons all over the world perform a vascular resection in cases of locally advanced PDAC in order to achieve R0 margins. Most data in the literature come from retrospective comparative studies; therefore, it is still unclear if such an aggressive approach to an advanced cancer is justified.

In our study, we retrospectively reviewed a prospective series of 173 patients with PDAC, 41 subjects underwent pancreatectomy with vascular resection for locally advanced disease, and in 132 patients, only a pancreatic resection was undertaken. Patients’ characteristics and operative and postoperative data were similar between groups. The operative time as a consequence of the additional procedure performed was significantly longer in the VR+ group. Despite a higher morbidity in the VR+ patients, there was no significant difference in length of stay and mortality between groups. Preoperative biliary drainage was necessary in a higher number of VR+ cases, and this may be responsible for the significantly increased complication rate in this group of patients [25]. A higher incidence of complications after biliary drainage has been observed also in two recent meta-analyses [26, 27]; however, the delay in surgery related to the stenting procedure does not seem to affect overall survival [28, 29]. Furthermore, other meta-analyses have found opposite results. Sun et al. [30] reported no significant difference in postoperative morbidity and mortality in patients with obstructive jaundice who underwent preoperative biliary stenting. Surprisingly, Moole et al. [31] observed significantly less major adverse events in patients undergoing preoperative biliary drainage compared to those undergoing direct surgery. In all these meta-analyses, the majority of data come from retrospective studies and results should be regarded with caution. The role of preoperative biliary drainage on postoperative outcomes probably needs to be further investigated in future studies.

In most series, only about two-thirds of patients who underwent vascular resection had a histologically proven invasion, whereas in our material this rate was slightly higher with 78% of patients with confirmed vessel wall infiltration. However, the presence of histological proven venous wall invasion in patients undergoing PD for pancreatic cancer has been reported to affect disease-free and overall survival with conflicting results [32–35]. Of note is that also the depth of wall invasion does not seem to influence survival rates. Roch et al. [36] analysed a series of 567 patients who underwent PD for PDAC, and segmental vein resection was performed in 90 cases. The extent of venous wall infiltration did not significantly influence overall survival and disease-free survival. Moreover, in a report from Hoshimoto et al. [37], the depth of vessel invasion did not impact overall survival in a series of 122 pancreatic cancer patients. In another study from Ravikumar et al. [38], a series of 229 patients undergoing PD with portal vein resection was analysed and no significant difference in median survival in patients with superficial, deep, or no histological involvement was observed. Finally, Addeo et al. [39] evaluated retrospectively 181 PDs and venous resection was performed in 91 cases. There was no difference in survival between patients with or without venous infiltration, but invasion of the intima was found to be an independent predictor of poor survival.

Previous research found that larger tumour size is correlated with reduced overall survival [10, 40]. Greater tumours are more likely associated to perineural infiltration and lymph node involvement that can explain a more aggressive behaviour. In our series, patients who underwent vascular resection had a significant larger tumour size and higher perineural infiltration rate, and this may justify the presence of venous/arterial wall invasion that required a more complex operation with a prolonged operating time. We also found in the VR+ cases a trend toward more frequent lymphovascular invasion and R1 resection, but these parameters were not statistically significant. Despite these aggressive features in the VR+ group, overall survival and long-term survival rates were comparable in the two groups, and these findings are similar to what was reported by others [20–24, 41].

Arterial infiltration (hepatic artery, celiac trunk, superior mesenteric artery) in patients with advanced PDAC is usually considered a contraindication for surgical resection because of increased morbidity and mortality [42, 43]. In fact, early in our experience, the four patients who underwent arterial resection had a much lower median survival compared to those undergoing isolated venous resection, though not significant. A very recent review from the French National Institute of Cancer [44] suggested that arterial resection may be proposed in selected patients only after response to neoadjuvant treatment.

The limitations associated with the present study include its retrospective nature and a limited number of patients. However, this is a single-centre series of consecutive patients who were operated by the same experienced hepatobiliary surgeon and they all received the same postoperative care protocol. Another limit is the absence of preoperative chemotherapy, but our survival rates were comparable between groups particularly if we consider patients who underwent isolated venous resection. Therefore, despite a higher morbidity, upfront surgery in selected patients with vascular involvement can be offered to improve survival.

## Conclusions

Our study confirms that pancreatectomy with vascular resection for a locally advanced PDAC is a complex operation associated with a significant longer operating time that may increase morbidity; however, in selected patients, R0 margins can be obtained with an acceptable long-term survival rate. Older patients are less likely to benefit from surgery. Further studies are warranted to better define the criteria for patient selection.

## Data Availability

The datasets used and/or analysed during the current study are available from the corresponding author on reasonable request.

## Abbreviations

PDAC: Pancreatic ductal adenocarcinoma
NCCN: National Comprehensive Cancer Network
BRPC: Borderline resectable pancreatic cancer
BMI: Body mass index
ASA: American Society of Anesthesiology
CT: Computed tomography
TNM: Tumour-Node-Metastasis
ISGPF: International Study Group for Pancreatic Surgery
POPF: Postoperative pancreatic fistula
ICU: Intensive care unit
HR: Hazard ratio
CI: Confidence interval
PD: Pancreaticoduodenectomy
